# Does Chronic Obstructive Pulmonary Disease Impact Outcome after Coronary Artery Bypass Grafting? A Population-Based Retrospective Study in Germany

**DOI:** 10.3390/jcm13175131

**Published:** 2024-08-29

**Authors:** Nadine Hochhausen, Marjolijn C. Sales, Natasja W. M. Ramnath, Sebastian Billig, Felix Kork, Ajay Moza

**Affiliations:** 1 Department of Anesthesiology, Medical Faculty, RWTH Aachen University, Pauwelsstrasse 30, 52074 Aachen, Germany; sebillig@ukaachen.de (S.B.); fkork@ukaachen.de (F.K.); 2 Department of Cardiac Surgery, Medical Faculty, RWTH Aachen University, Pauwelsstrasse 30, 52074 Aachen, Germany; msales@ukaachen.de (M.C.S.); nramnath@ukaachen.de (N.W.M.R.); amoza@ukaachen.de (A.M.)

**Keywords:** chronic obstructive pulmonary disease, coronary artery bypass grafting, off-pump coronary artery bypass surgery, in-hospital mortality

## Abstract

**Background:** The interaction between chronic obstructive pulmonary disease (COPD) and coronary artery bypass grafting (CABG) is discussed controversial. **Methods:** In this population-based retrospective analysis including non-emergency CABG in Germany between 2015 and 2021, the aim was to compare in-hospital mortality, hospital length of stay (HLOS), and perioperative ventilation time (VT) in patients affected by COPD and not affected by COPD. In addition, we compared outcomes after off-pump coronary artery bypass (OPCAB) and on-pump coronary artery bypass (ONCAB) surgery and outcomes after CABG with a minimally invasive technique with and without cardiopulmonary bypass (CPB) in COPD patients. **Results:** Of the 274,792 analyzed cases undergoing non-emergency CABG, 7.7% suffered from COPD. COPD patients showed a higher in-hospital mortality (6.0% vs. 4.2%; *p* < 0.001), a longer HLOS (13 days (10–19) vs. 12 days (9–16); *p* < 0.001), and a longer VT (33 h (11–124) vs. 28 h (9–94); *p* < 0.001). In subgroup analyses, COPD patients undergoing OPCAB surgery showed a lower in-hospital mortality (3.5% vs. 6.4%; *p* < 0.001), a shorter HLOS (12 days (9–16) vs. 13 days (10–19); *p* < 0.001) and a shorter VT (20 h (10–69) vs. 36 h (11–135); *p* < 0.001) compared to ONCAB surgery. Regression analyses confirmed that using cardiopulmonary bypass in COPD patients is associated with a higher risk of in-hospital mortality (OR, 1.86; 95% CI: 1.51–2.29, *p* < 0.001), a longer HLOS (1.44 days; 95% CI: 0.91–1.97, *p* < 0.001), and a longer VT (33.67 h; 95% CI: 18.67–48.66, *p* < 0.001). In further subgroup analyses, COPD patients undergoing CABG with a minimally invasive technique without CPB showed a lower in-hospital mortality (3.5% vs. 16.5%; *p* < 0.001) and a shorter VT (20 h (10–69) vs. 65 h (29–210); *p* < 0.001) compared to CABG with a minimally invasive technique and CPB. Regression analyses confirmed that using CPB in COPD patients undergoing CABG with a minimally invasive technique is associated with a higher risk of in-hospital mortality (OR, 4.80; 95% CI: 2.42–9.51, *p* < 0.001). **Conclusions:** COPD negatively impacts outcomes after non-emergency CABG. According to our results, OPCAB surgery and CABG with a minimally invasive technique without CPB seem to be beneficial for COPD patients. Further studies should be performed to confirm this.

## 1. Introduction

Coronary artery bypass grafting (CABG) has been established as a standard-of-care treatment in coronary artery disease (CAD) for patients with diffuse or multivessel CAD, left ventricular dysfunction, or left main coronary artery involvement [1,2]. Conventional CABG is performed using a cardiopulmonary bypass (on-pump coronary artery bypass; ONCAB) [3,4]. ONCAB surgery is still common [5], even though less invasive procedures are becoming more frequent. A commonly performed less invasive procedure is off-pump coronary artery bypass (OPCAB) surgery [6]. OPCAB surgery has been shown to be beneficial in high-risk patients [2]. If surgical revascularization of an isolated stenosis of the left descending artery is necessary, CABG with a minimally invasive technique, e.g., minimally invasive direct CABG (MIDCAB) surgery, can be performed [2].

It is well known that CAD is frequently associated with chronic obstructive pulmonary disease (COPD) [7]. COPD is characterized by chronic obstructive bronchitis and/or emphysema based on a progressive airway disease [7,8]. It is a known fact that COPD patients show worse perioperative outcomes than patients not suffering from COPD [9]. However, previous reports on outcomes including COPD patients undergoing CABG [10,11] and OPCAB surgery [12,13,14] have not been consistent.

We hypothesized that COPD negatively impacts the outcome of patients undergoing non-emergency CABG. In addition, we hypothesized that OPCAB surgery is beneficial in COPD patients compared to ONCAB surgery. Therefore, we carried out a population-based retrospective cohort study in patients undergoing non-emergency CABG. Here, we evaluated the impact of COPD on different outcome parameters, such as in-hospital mortality, hospital length of stay (HLOS), and perioperative ventilation time (VT). 

## 2. Materials and Methods

### 2.1. Source of Data

We analyzed de-identified data that were made accessible via remote data processing. The actual data were not accessible to the authors. For that reason, no institutional or review board approval was necessary.

In Germany, hospitals are obliged to use the case-based DRG system. Therefore, all in-patient hospital cases are recorded for reimbursement calculations and afterwards presented in an annual survey. Upon request, researchers are allowed to analyze these cases via remote data processing; we drafted an analysis protocol in a Stata do-file (Stata BE 17 for Windows, StataCorp, College Station, TX, USA) based on sample data structure files. Then, the Stata do-file was transmitted to the Federal Statistical Office where the analyses were performed on the actual data (Stata 15 for Windows, StataCorp, College Station, TX, USA). Hereafter, the de-identified and curated results were provided to the authors. Raw data were not accessible to the authors at any time. Our study used the German Diagnosis-Related Groups (G-DRG) Statistik (Source: RDC of the Federal Statistical Office and Statistical Offices of the Federal States, own calculations, Supplementary File S1) provided by the Federal Statistical Office of Germany. 

### 2.2. Inclusion and Exclusion Criteria

**Figure 1:**
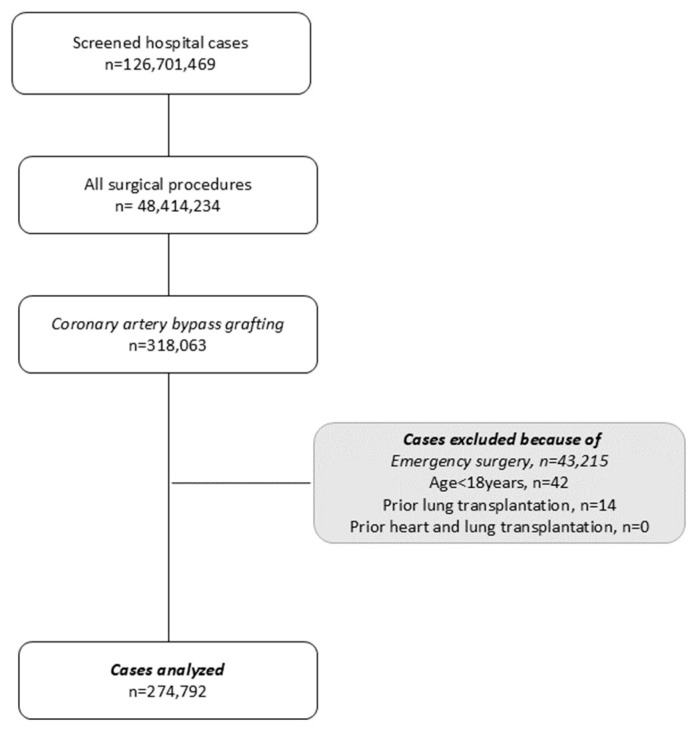
Flowchart of patient inclusion of a population-based retrospective study analyzing the impact of COPD on in-hospital mortality, hospital length of stay (HLOS), and ventilation time (VT) in 274,792 cases undergoing non-emergency coronary artery bypass grafting (CABG).

We included all patients undergoing non-emergency CABG in Germany between 1 January 2015, and 31 December 2021. We excluded patients younger than 18, those with a prior heart or lung transplantation, and cases that were admitted to hospital as emergencies (Figure 1).

### 2.3. Variables

CABG surgery data were obtained from the German procedure classification codes (Operationen-und Prozedurenschlüssel-OPS), which are a modified version of the International Classification of Procedures in Medicine (ICPM) as established by the World Health Organization (WHO). The procedure classification codes ‘aortocoronary bypass procedure’ (cardiopulmonary bypass (CPB) is included in this code) and ‘aortocoronary bypass procedure using a minimally invasive technique’ (using an additional code if CPB was used) were used for this study. Additionally, we identified the presence of COPD from the International Statistical Classification of Diseases and Related Health Problems, Tenth Revision (ICD-10). Further variables, such as age, sex, diagnoses, in-hospital mortality, HLOS, and VT were also extracted from ICD-10 diagnosis codes and OPS procedure codes. Moreover, the Charlson Comorbidity Index (CCI), as introduced by Quan et al. [15], was determined. The required comorbidities for determining the CCI, were also extracted from ICD-10 diagnosis codes and OPS procedure codes (Supplementary File S1).

### 2.4. Outcomes

We defined in-hospital mortality as the primary endpoint and HLOS and VT as secondary endpoints. In-hospital mortality, HLOS, and VT are actual data recorded in the G-DRG data; no further data transformation was conducted on these outcome variables.

### 2.5. Statistical Analyses

As this is a retrospective cohort study, we did not calculate the sample size or perform a power analysis. The Federal Statistical Office performed the analysis (Stata 15 for Windows, StataCorp, College Station, TX, USA) based on the analysis protocol coded by the authors. We considered statistical significance at *p* < 0.01. Continuous variables were stated as median and interquartile range (IQR) and compared using the Mann–Whitney U-test. In addition, frequencies were stated as numbers and percentages and compared using the Chi-squared test. Moreover, binary logistic regression models were used to estimate associations with in-hospital mortality, and robust regression models were used to estimate the associations with HLOS and VT. 

## 3. Results

### 3.1. Study Population

A total of 126,701,469 hospital cases were screened between 2015 and 2021. Surgery was performed in 48,414,234 cases, of which 318,063 cases were classified as CABG. Cases with under 18 years of age (n = 42), with prior lung transplantation (n = 14) and with prior heart-lung transplantation (n = 0) were excluded. Moreover, cases that were admitted as emergencies to the hospital for CABG (n = 43,215) were also excluded. The remaining 274,792 cases with non-emergency CABG were analyzed in this study (Figure 1).

Of these 274,792 cases, 231,334 (84.2%) were coded as ‘aortocoronary bypass procedure’. Here, using CPB is already included according to the procedures’ classification code. A total of 43,458 (15.8%) cases were coded as ‘aortocoronary bypass procedure using a minimally invasive technique’. No categorization into various access sites, such as sternotomy, thoracotomy or endoscopic access, was made. 

The characteristics of all cases analyzed undergoing non-emergency CABG are represented in Table 1. The median age of patients was 69 years (IQR, 62–76), and most patients were male (79.5%). Of the comorbidities analyzed, congestive heart failure (50.3%) was the most common, followed by myocardial infarction (33.0%) and uncomplicated diabetes mellitus (29.8%). The median CCI turned out to be 2 (IQR, 1–3). In-hospital mortality of non-emergency CABG was 4.3%, the median HLOS was 12 days (IQR, 9–16), and the median VT was 29 h (IQR, 10–96).

### 3.2. COPD and CABG

In our initial analysis, we compared patients with and without COPD undergoing CABG.

In total, 7.7% (n = 21,240) of the study population suffered from COPD (Table 1). COPD patients were slightly older (70 years (IQR, 62–76) vs. 69 years (62–76), *p* < 0.001) and a worse state of health was represented by a higher CCI (3 (IQR, 2–5) vs. 2 (IQR, 1–3), *p* < 0.001) than patients not suffering from COPD. Patients with and without COPD undergoing CABG were predominantly of male gender (78.9% vs. 79.6%, *p* = 0.045) and were most frequently affected by congestive heart failure (60.2% vs. 49.4%, *p* < 0.001), and hereafter by myocardial infarction (33.6% vs. 32.9%, *p* = 0.041) and uncomplicated diabetes mellitus (31.6% vs. 29.6%, *p* < 0.001) (Table 1).

#### Impact of COPD on In-Hospital Mortality, HLOS and VT in Patients Undergoing CABG

In-hospital mortality was higher in COPD patients compared to patients without COPD (6.0% vs. 4.2%, *p* < 0.001). Moreover, a longer median HLOS (13 days (IQR, 10–19) vs. 12 days (IQR, 9–16), *p* < 0.001) was observed in patients with COPD compared to patients without COPD. Moreover, COPD patients were more often ventilated (42.9% vs. 32.5%) and demonstrated a longer VT (33 h (IQR, 11–124) vs. 28 h (IQR, 9–94), *p* < 0.001) than patients without COPD (Table 1). 

### 3.3. COPD in OPCAB and ONCAB Surgery

After demonstrating that COPD was associated with adverse outcome, we analyzed COPD patients undergoing OPCAB compared to ONCAB surgery to shed more light on the role of CPB in COPD patients.

In COPD patients, OPCAB surgery was performed in 2986 cases (14.1%), and ONCAB surgery was performed in 18,254 cases (85.9%) (Table 2). The median age was 70 years in OPCAB and ONCAB surgery (both, 70 years (IQR, 63–76), *p* = 0.314), and most of the patients were male (80.0 vs. 78.9, *p* = 0.314). The median CCI turned out to be 3 (IQR, 2–5), regardless of whether OPCAB or ONCAB was performed. Differences in comorbidities represented by the CCI was solely observed in congestive heart failure (57.2 vs. 60.6, *p* < 0.001), cerebrovascular disease (16.0 vs. 19.5, *p* < 0.001) and hemiplegia or paraplegia (2.4 vs. 3.6, *p* = 0.001); these co-morbidities were observed less frequently in patients undergoing OPCAB surgery (Table 2).

#### Impact of COPD on In-Hospital Mortality, HLOS and VT in Patients with OPCAB and ONCAB Surgery

Considering COPD patients, in-hospital mortality was lower (3.5% vs. 6.4%, *p* < 0.001) and median HLOS (12 days (IQR, 9–16) vs. 13 days (IQR, 10–19), *p* < 0.001) was reduced in OPCAB compared to ONCAB surgery.

In addition, the need for perioperative ventilation was lower (37.3% vs. 43.8%) and the median VT (20 h (IQR, 10–69) vs. 36 h (IQR, 11–135), *p* < 0.001) was reduced in patients undergoing OPCAB compared to ONCAB surgery (Table 2).

### 3.4. COPD in CABG Using a Minimally Invasive Technique with and without CPB

Finally, and to rule out any potential confounding effects of different surgical trauma, we analyzed COPD patients undergoing CABG with a minimally invasive technique with and without CPB.

Regarding COPD patients, CABG using a minimally invasive technique without CPB was performed in 2986 (97.4%) cases and minimally invasive technique with CPB was performed in 79 cases (2.6%). The median age of patients undergoing a minimally invasive procedure without CPB was 70 years (IQR, 63–76), compared to 66 years (IQR, 61–76) in patients undergoing a minimally invasive procedure with CPB (*p* = 0.224). The majority of patients were male (80% vs. 72.2%, *p* = 0.088). The median CCI turned out to be 3 (IQR, 2–5) in patients undergoing a minimally invasive procedure without CPB, and 4 (IQR, 3–5) in patients undergoing a minimally invasive procedure with CPB (*p* < 0.001). Concerning the comorbidities, a statistical difference between both groups could only be observed in peripheral vascular disease (32.4% vs. 57.0%, *p* < 0.001) (Table 3).

#### Impact of COPD on In-Hospital Mortality, HLOS and VT in Patients Undergoing CABG with a Minimally Invasive Technique with and without CPB

In COPD patients, in-hospital mortality was lower when CABG with a minimally invasive technique without CPB was performed compared to CABG with a minimally invasive technique with CPB (3.5% vs. 16.5%, *p* < 0.001). Moreover, the need for ventilation (37.3% vs. 53.2%) and the median VT were lower when CABG with a minimally invasive technique without CPB was performed (20 h (IQR, 10–69) vs. 65 h (IQR, 29–210), *p* < 0.001). Regarding the median HLOS, no statistical difference could be observed between groups (12 days (IQR, 9–16) vs. 13 days (IQR, 9–20), *p* = 0.661).

### 3.5. Regression Analyses

Table 4 provides a summary of the most important results of regression analyses. The detailed findings are made available as Supplementary Files (Supplementary Files S2–S16).

#### 3.5.1. Regression Analysis: In-Hospital Mortality

No higher risk of in-hospital mortality was associated with COPD (odds ratio (OR), 0.94; 95% confidence interval (CI): 0.84–1.04, *p* = 0.214), when undergoing non-emergency CABG. 

In patients with and without COPD, using CPB was associated with a higher odds for in-hospital mortality (patients with COPD: OR, 1.86; 95% CI: 1.51–2.29, *p* < 0.001; patients without COPD: OR, 2.19; 95% CI: 2.03–2.37, *p* < 0.001).

Moreover, using CPB with a minimally invasive technique was associated with a higher odds for in-hospital mortality in patients with and without COPD (patients with COPD: OR, 4.80; 95% CI: 2.42–9.51, *p* < 0.001; patients without COPD: OR, 6.73; 95% CI: 5.50–8.25, *p* < 0.001) (Table 4). 

#### 3.5.2. Regression Analysis: HLOS

COPD was associated with a reduced HLOS (beta, −0.73 days; 95% CI: −1.07–−0.38, *p* < 0.001), when undergoing non-emergency CABG.

Moreover, a longer HLOS was demonstrated for the use of CPB with and without COPD (COPD: beta, 1.44 days; 95% CI: 0.91–1.97; *p* < 0.001 and no-COPD: beta, 1.61 days; 95% CI: 1.51–1.71; *p* < 0.001). 

No statistical significance was shown for a longer HLOS when CPB with a minimally invasive technique was used in patients with COPD, but significance did emerge for patients without COPD (COPD: beta, 1.03 days; 95% CI: −2.13–4.19; *p* = 0.522 and no-COPD: beta, 3.21 days; 95% CI: 2.33–4.10; *p* < 0.001) (Table 4).

#### 3.5.3. Regression Analysis: VT

Additionally, COPD was also associated with a reduced VT (beta, −24.32 h, 95% CI: −34.46–−14.17, *p* < 0.001) when undergoing non-emergency CABG. 

The use of CPB was associated with a longer VT in patients with and without COPD (COPD: 33.67 h, 95% CI: 18.67–48.66, *p* < 0.001 and No-COPD: 30.78 h, 95% CI: 26.45–35.11, *p* < 0.001). 

No statistical significance for a longer VT was demonstrated when CPB with a minimally invasive technique was used in patients with COPD, but significance did emerge for patients without COPD (COPD: beta, 51.88 h; 95% CI: −23.66–127.42; *p* = 0.178 and no-COPD: beta, 111.80 h; 95% CI: 87.85–135.76; *p* < 0.001) (Table 4).

### 3.6. Regression Analysis: Confounders

Different confounders were addressed in the regression analyses. The analyses of the regression models showed that moderate-to-severe liver disease (OR, 10.76; 95% CI: 9.08–12.76, *p* < 0.001) is the most important risk factor for in-hospital mortality (Supplementary File S2) in CABG. 

When considering COPD patients, mild (OR, 2.54; 95% CI: 1.97–3.27, *p* < 0.001) and moderate-to-severe liver disease (OR, 5.51; 95% CI: 3.10–9.80, *p* < 0.001) and chronic heart failure (OR, 2.58; 95% CI: 2.23–2.99, *p* < 0.001) are the most important risk factors for in-hospital mortality (Supplementary File S5).

## 4. Discussion

In this retrospective study of patients undergoing non-emergency CABG between 2015 and 2021, we first examined whether COPD patients had a worse outcome as defined by higher in-hospital mortality, longer HLOS, and longer VT compared to patients without COPD. Secondly, we analyzed and compared the outcome of COPD patients undergoing OPCAB and ONCAB surgery. Thirdly, we analyzed and compared the outcome of COPD patients undergoing CABG with a minimally invasive technique with and without CPB. In 274,792 CABG cases, COPD patients showed a higher in-hospital mortality and a longer HLOS as well as a longer VT compared to patients without COPD in univariate analysis. Regression analyses could not demonstrate a higher risk of in-hospital mortality, but did show a shorter HLOS and a shorter VT in COPD patients. A subgroup analysis showed a lower in-hospital mortality, a shorter HLOS, and a shorter VT in COPD patients undergoing OPCAB surgery compared to ONCAB surgery. Regression analyses confirmed these findings. A further subgroup analysis showed a lower in-hospital mortality and a shorter VT in COPD patients undergoing CABG with a minimally invasive technique without CPB compared to the use of CPB. However, no statistical significance was reached for HLOS between groups. Regression analyses could demonstrate a higher risk of in-hospital mortality, but not a shorter HLOS or VT.

There are conflicting results regarding the impact of COPD on outcome after CABG. Some investigations did not demonstrate a difference in in-hospital mortality after CABG in patients with and without COPD [16,17]. Other studies reported a higher mortality or increased postoperative complication in COPD patients [18,19]. In addition, the SYNTAX Extended Survival Study reported higher long-term mortality in COPD patients [20]. Differences in the outcome of COPD patients after CABG may be due to various patient selections, e.g., different access sites and comorbidities. Different definitions of COPD, different patient populations, or different coding systems could also be responsible for varying results. Our study demonstrated higher in-hospital mortality, a longer HLOS and a longer VT in COPD patients. However, regression model analysis was not able to confirm that COPD is associated with a higher risk of in-hospital mortality. Surprisingly, COPD in CABG was associated with a slightly reduced HLOS and VT in regression analyses. One possible explanation is that COPD patients receive increased attention in perioperative ventilation management, leading to an earlier transfer to a rehabilitation unit or to a specialized respiratory ward. Another possible explanation is that mild COPD is underdiagnosed causing a ‘falsification’ of the results. Nevertheless, we clearly demonstrated the impact of COPD on CABG outcomes. Regarding less invasive CABG procedures, OPCAB surgery is considered to have advantages over ONCAB surgery [21,22,23,24]. OPCAB surgery has shown the benefits of reducing the incidence of transfusion requirements [23] and perioperative myocardial infarction, major complications, intensive care unit stays, and mortality [24]. Kuss et al. extended the evaluation by including red blood cell transfusion and mortality by stroke, renal failure, wound infection, prolonged ventilation, inotropic support and intra-aortic balloon pump support [22]. However, the ROOBY trial sub-study reported that intraoperative complications were higher in COPD patients undergoing OPCAB compared to ONCAB surgery [25]. The higher incidence of ventricular fibrillation and cardiac arrest in COPD patients was attributed to an increased probability of hemodynamic instability, a longer operation time, and the necessity of ventilatory changes during heart positioning [25]. Our findings demonstrated that in-hospital mortality was lower in COPD patients undergoing OPCAB surgery. Additionally, HLOS and VT were reduced in OPCAB surgery. Moreover, we could demonstrate that in-hospital mortality was lower, and VT was shorter in COPD patients undergoing CABG with a minimally invasive technique without CPB compared to CABG with a minimally invasive technique with CPB.

Our findings indicate that the impact of COPD on CABG outcomes should alert clinicians to the need for careful management of these patients. One approach is to focus on optimizing preoperative COPD therapy as a preventive measure to enhance outcome. Additionally, individualized perioperative ventilation strategies could positively affect outcomes. Furthermore, our findings suggest that COPD patients benefit from OPCAB surgery. Therefore, whenever possible, OPCAB surgery and CABG with a minimally invasive technique without CPB should be considered for COPD patients.

Our retrospective cohort study presents various strengths and limitations that should be discussed. First, the main strength is the cohort size: we analyzed 274,792 cases undergoing non-emergency CABG between 2015 and 2021 in Germany. Due to the large study population, our results must be considered reliable, even if the likelihood of errors occurring is higher in large data studies. Second, the requirement of thorough documentation in Germany for reimbursement purposes further supports the reliability of our results. However, the possibility of inadequate coding cannot be entirely excluded, particularly regarding the classification of COPD severity grades, which may lead to coding errors. As a result, we did not classify the COPD patient population in severity grades, which is a limitation. Additionally, the CCI does not accurately represent the comorbidities of cardiac surgery patients. However, due to the retrospective nature of our study, no other data were available. The main limitations are that we did not analyze other risk factors influencing the cardiopulmonary system, such as cigarette smoking, and that we did not consider the access site. In addition, the severity of the disease (single or multivessel revascularization, type of grafts, etc.) was also not considered. Further deviating and interesting results could be expected if these parameters are considered. Moreover, the ONCAB group may include OPCAB surgery due to the nature of the coding system. However, it is highly likely that these account for only a minority. Nevertheless, we performed a further subgroup analysis, which considered COPD patients undergoing CABG with a minimally invasive technique with and without CPB. However, the results may differ if more detailed categorization is applied. Another limitation is that we analyzed data from only one country. Variations in hospitalization period and postoperative management practice in other countries could influence and limit the generalizability of our finding. Moreover, we did not consider re-admission to the hospital or perioperative complications, such as conversion, reopening for bleeding, transfusion, etc., in this study. These confounders may also affect in-hospital mortality, HLOS, and VT. Future studies should focus on hospital re-admissions and long-term mortality. If long-term mortality is considered, a deviation from the current results would be possible. In addition, a deviation from the current results could be expected if another patient selection was performed.

## 5. Conclusions

Upon univariate analysis, this study showed higher in-hospital mortality, a longer HLOS, and a longer VT in COPD patients undergoing CABG. However, these findings were not confirmed by regression analyses. Additionally, the study demonstrated that COPD patients undergoing OPCAB surgery had lower in-hospital mortality, shorter HLOS, and shorter VT compared to those undergoing ONCAB surgery, as confirmed by regression analyses. Moreover, a lower in-hospital mortality and a shorter VT was demonstrated in COPD patients undergoing a minimally invasive procedure without CPB. Regression analysis could only confirm a higher risk of in-hospital mortality when CPB is used with a minimally invasive technique. To validate these results, prospective investigations should be conducted, with a particular emphasis on confounders. Furthermore, future studies should focus on intraoperative complications.

## Figures and Tables

**Table 1:** Characteristics and outcome of 274,792 patients affected by chronic obstructive pulmonary disease (COPD) and not affected by COPD undergoing non-emergency coronary artery bypass grafting (CABG).

Characteristic	All Patients (n = 274,792)	Patients with COPD (n = 21,240; 7.7%)	Patients without COPD (n = 253,552; 92.3%)	*p*-Value
***Sociodemographic characteristics***				
Median age (IQR) years	69 (62–76)	70 (63–76)	69 (62–76)	<0.001
Gender				
Female-no (%)	56,304 (20.5)	4488 (21.1)	51,813 (20.4)	0.045
Male-no (%)	218,483 (79.5)	16,752 (78.9)	201,731 (79.6)	0.045
Unknown-no (%)	5 (0.0)	0 (0.0)	5 (0.0)	0.045
Median Charlson comorbidity index (IQR)-pts	2 (1–3)	3 (2–5)	2 (1–3)	<0.001
***Charlson Comorbidity Index Items-no (%)***				
Myocardial infarction	90,568 (33.0)	7135 (33.6)	83,433 (32.9)	0.041
Congestive heart failure	138,114 (50.3)	12,778 (60.2)	125,336 (49.4)	<0.001
Peripheral vascular disease	58,929 (21.4)	6830 (32.2)	52,099 (20.5)	<0.001
Cerebrovascular disease	40,148 (14.6)	4044 (19.0)	36,104 (14.2)	<0.001
Dementia	1405 (0.5)	142 (0.7)	1263 (0.5)	0.001
Chronic pulmonary disease	31,227 (11.4)	21,240 (100.0)	9987 (3.9)	<0.001
Rheumatoid disease	3201 (1.2)	298 (1.4)	2903 (1.1)	0.001
Peptic ulcer disease	1538 (0.6)	169 (0.8)	1369 (0.5)	<0.001
Liver disease				
Mild	4974 (1.8)	595 (2.8)	4379 (1.7)	<0.001
Moderate-to-severe	665 (0.2)	62 (0.3)	603 (0.2)	0.123
Diabetes mellitus				
Uncomplicated	81,859 (29.8)	6704 (31.6)	75,155 (29.6)	<0.001
With end-organ damage	15,060 (5.5)	1507 (7.1)	13,553 (5.3)	<0.001
Hemiplegia or paraplegia	7975 (2.9)	727 (3.4)	7248 (2.9)	<0.001
Renal disease	50,361 (18.3)	5165 (24.3)	45,196 (17.8)	<0.001
Cancer				
Non-metastatic	3189 (1.2)	340 (1.6)	2849 (1.1)	<0.001
Metastatic	375 (0.1)	30 (0.1)	345 (0.1)	0.844
AIDS	84 (0.03)	2 (0.0)	82 (0.0)	0.066
***Outcome parameter***				
In-hospital mortality-no (%)	11,798 (4.3)	1269 (6.0)	10,529 (4.2)	<0.001
Median hospital length of stay days (IQR)	12 (9–16)	13 (10–19)	12 (9–16)	<0.001
Median ventilation time-hours (IQR)	29 (10–96) n = 91,611 (33.3%)	33 (11–124) n = 9103 (42.9%)	28 (9–94) n = 82,508 (32.5%)	<0.001

no: number, IQR: interquartile range.

**Table 2:** Characteristics and outcome of 274,792 patients affected by chronic obstructive pulmonary disease (COPD) and not affected by COPD undergoing off-pump coronary artery bypass (OPCAB) and on-pump coronary artery bypass (ONCAB) surgery.

	COPD (n = 21,240)			No COPD (n = 253,552)		
Characteristic	OPCAB (n = 2986; 14.1%)	ONCAB (n = 18,254; 85.9%)	*p*-Value	OPCAB (n = 39,368; 15.5%)	ONCAB (n = 214,184; 84.5%)	*p*-Value
	***Sociodemographic characteristics***					
Median age (IQR) years	70 (63–76)	70 (63–76)	0.314	69 (61–76)	69 (62–76)	<0.001
Gender						
Female-number (%)	598 (20.0)	3890 (21.3)	0.111	XXX	XXX	XXX
Male-number (%)	2388 (80.0)	14,364 (78.9)	0.111	31,597 (80.3)	170,134 (79.4)	<0.001
Median Charlson comorbidity index (IQR)-points	3 (2–5)	3 (2–5)	0.0001	2 (1–3)	2 (1–3)	<0.001
	***Charlson Comorbidity Index Items-number (%)***					
Myocardial infarction	975 (32.7)	6160 (33.7)	0.241	12,364 (31.4)	71,069 (33.2)	<0.001
Congestive heart failure	1709 (57.2)	11,069 (60.6)	<0.001	17,415 (44.2)	107,921 (50.4)	<0.001
Peripheral vascular disease	968 (32.4)	5862 (32.1)	0.741	6727 (17.1)	45,372 (21.2)	<0.001
Cerebrovascular disease	477 (16.0)	3567 (19.5)	<0.001	4456 (11.3)	31,648 (14.8)	<0.001
Dementia	13 (0.4)	129 (0.7)	0.092	167 (0.4)	1096 (0.5)	0.023
Chronic pulmonary disease	2986 (100.0)	18,254 (100.0)	n/a	1303 (3.3)	8684 (4.1)	<0.001
Rheumatoid disease	40 (1.3)	258 (1.4)	0.751	354 (0.9)	2549 (1.2)	<0.001
Peptic ulcer disease	26 (0.9)	143 (0.8)	0.618	140 (0.4)	1229 (0.6)	<0.001
Liver disease						
Mild	78 (2.6)	517 (2.8)	0.499	528 (1.3)	3851 (1.8)	<0.001
Moderate-to-severe	XXX	XXX	0.014	49 (0.1)	554 (0.3)	<0.001
Diabetes mellitus						
Uncomplicated	936 (31.3)	5768 (31.6)	0.783	11,385 (28.9)	63,770 (29.8)	0.001
With end-organ damage	215 (7.2)	1292 (7.0)	0.809	2088 (5.3)	11,465 (5.4)	0.691
Hemiplegia or paraplegia	72 (2.4)	655 (3.6)	0.001	708 (1.8)	6540 (3.1)	<0.001
Renal disease	699 (23.4)	4466 (24.5)	0.212	5694 (14.5)	39,232 (18.3)	<0.001
Cancer						
Non-metastatic	55 (1.8)	285 (1.6)	0.257	373 (0.9)	2476 (1.2)	<0.001
Metastatic	7 (0.2)	23 (0.1)	0.144	53 (0.1)	292 (0.1)	0.933
AIDS	XXX	XXX	XXX	13 (0.03)	69 (0.03)	0.935
	***Outcome parameter***					
In-hospital mortality-number (%)	104 (3.5)	1165 (6.4)	<0.001	748 (1.9)	9781 (4.6)	<0.001
Median hospital length of stay days (IQR)	12 (9–16)	13 (10–19)	<0.001	11 (8–14)	12 (9–16)	<0.001
Median ventilation time-hours (IQR)	20 (10–69) n = 1115 (37.3%)	36 (11–135) n = 7988 (43.8%)	<0.001	15 (8–38) n = 9969 (25.3%)	30 (10–101) n = 72,539 (33.9%)	<0.001

IQR: interquartile range. XXX: For reasons of data protection, this number was not published.

**Table 3:** Characteristics and outcomes of 43,458 patients affected by chronic obstructive pulmonary disease (COPD) and not affected by COPD undergoing an aortocoronary bypass procedure (CABG) using a minimally invasive technique with and without cardiopulmonary bypass (CPB).

	COPD (n = 3065)			No COPD (n = 40,393)		
Characteristic	CABG Using a Minimally Invasive Technique without CPB (n = 2986; 97.4%)	CABG Using a Minimally Invasive Technique with CPB (n = 79; 2.6%)	*p*-Value	CABG Using a Minimally Invasive Technique without CPB (n = 39,368; 97.5%)	CABG Using a Minimally Invasive Technique with CPB (n = 1025; 2.5%)	*p*-Value
	***Sociodemographic characteristics***					
Median age (IQR) years	70 (63–76)	66 (61–76)	0.224	69 (61–76)	68 (60–76)	0.229
Gender						
Female-number (%)	598 (20.0)	22 (27.8)	0.088	XXX	XXX	XXX
Male-number (%)	2388 (80.0)	57 (72.2)	0.088	XXX	XXX	XXX
Median Charlson comorbidity index (IQR)-points	3 (2–5)	4 (3–5)	<0.001	2 (1–3)	2 (1–3)	<0.001
	***Charlson Comorbidity Index Items-number (%)***					
Myocardial infarction	975 (32.7)	39 (49.4)	0.002	12,364 (31.4)	475 (46.3)	<0.001
Congestive heart failure	1709 (57.2)	53 (67.1)	0.080	17,415 (44.2)	608 (59.3)	<0.001
Peripheral vascular disease	968 (32.4)	45 (57.0)	<0.001	6727 (17.1)	289 (28.2)	<0.001
Cerebrovascular disease	477 (16.0)	18 (22.8)	0.104	4456 (11.3)	185 (18.0)	<0.001
Dementia	13 (0.4)	0 (0)	0.557	167 (0.4)	10 (1.0)	0.008
Chronic pulmonary disease	2986 (100)	79 (100)	n/a	1303 (3.3)	33 (3.2)	0.873
Rheumatoid disease	40 (1.3)	XXX	XXX	354 (0.9)	14 (1.4)	0.121
Peptic ulcer disease	26 (0.9)	0 (0)	0.405	140 (0.4)	11 (1.1)	<0.001
Liver disease						
Mild	78 (2.6)	XXX	XXX	528 (1.3)	17 (1.7)	0.385
Moderate-to-severe	XXX	XXX	XXX	49 (0.1)	3 (0.3)	0.138
Diabetes mellitus						
Uncomplicated	936 (31.3)	25 (31.6)	0.955	11,385 (28.9)	283 (27.6)	0.361
With end-organ damage	215 (7.2)	11 (13.9)	0.024	2088 (5.3)	66 (6.4)	0.110
Hemiplegia or paraplegia	72 (2.4)	3 (3.8)	0.431	708 (1.8)	46 (4.5)	<0.001
Renal disease	699 (23.4)	20 (25.3)	0.693	5964 (14.5)	172 (16.8)	0.151
Cancer						
Non-metastatic	55 (1.8)	XXX	XXX	373 (0.9)	6 (0.6)	0.235
Metastatic	7 (0.2)	0 (0)	0.667	53 (0.1)	3 (0.3)	0.179
AIDS	0	0 (0)	n/a	13 (0.03)	0 (0)	0.561
In-hospital mortality-number (%)	104 (3.5)	13 (16.5)	<0.001	748 (1.9)	147 (14.3)	<0.001
Median hospital length of stay days (IQR)	12 (9–16)	13 (9–20)	0.661	11 (8–14)	13 (9–19)	<0.001
Median ventilation time-hours (IQR)	20 (10–69) n = 1115 (37.3%)	65 (29–210) n = 42 (53.2%)	<0.001	15 (8–38) n = 9969 (25.3%)	89 (28–291) n = 485 (47.3%)	<0.001

IQR: interquartile range. XXX: For reasons of data protection, this number was not published. n/a: not applicable.

**Table 4:** Risk-adjusted associations from multivariable regression analyses models on coronary artery bypass grafting (CABG) for in-hospital mortality, hospital length of stay (HLOS), and perioperative ventilation time (VT).

	In-Hospital Mortality		Hospital Length of Stay		Ventilation Time	
	Odds Ratio (95% CI)	*p*-Value	Beta (95% CI)	*p*-Value	Beta (95% CI)	*p*-Value
**Study population**						
COPD in patients undergoing CABG	0.94 (0.84– 1.04)	0.214	−0.73 (−1.07–−0.38)	<0.001	−24.32 (−34.46–−14.17)	<0.001
**Subgroup analysis**						
Use of cardiopulmonary bypass in patients with COPD	1.86 (1.51– 2.29)	<0.001	1.44 (0.91–1.97)	<0.001	33.67 (18.67–48.66)	<0.001
Use of cardiopulmonary bypass in patients without COPD	2.19 (2.03– 2.37)	<0.001	1.61 (1.51–1.71)	<0.001	30.78 (26.45–35.11)	<0.001
Use of cardiopulmonary bypass undergoing CABG using a minimally invasive technique in patients with COPD	4.80 (2.42– 9.51)	<0.001	1.03 (−2.13–4.19)	0.522	51.88 (−23.66–127.42)	0.178
Use of cardiopulmonary bypass undergoing CABG using a minimally invasive technique in patients without COPD	6.73 (5.50– 8.25)	<0.001	3.21 (2.33–4.10)	<0.001	111.80 (87.85–135.76)	<0.001

## Data Availability

Data are contained within the article or Supplementary Materials.

## Supplementary Materials

The following supporting information can be downloaded at: https://www.mdpi.com/article/10.3390/jcm13175131/s1.
