# Supplementary material for: Does Chronic Obstructive Pulmonary Disease Impact Outcome after Coronary Artery Bypass Grafting? A Population-Based Retrospective Study in Germany
Source: J Clin Med. 2024 Aug 29;13(17):5131. doi: 10.3390/jcm13175131 (PMC11396234; doi:10.3390/jcm13175131)
Supplement: Supplementary file 1 [file jcm-13-05131-s001.zip › Additional File 10_Regression_ no copd_on pump_VT.pdf]

Additional File 10. Risk-Adjusted associations of **perioperative ventilation time** from multivariable regression analysis models analyzing the impact of on-pump aorto-coronary bypass surgery in 82,508 patients not suffering from chronic obstructive pulmonary disease (no-COPD).

	**Coefficient (95% CI)**	**P- value**
**On-pump surgery**	30.78 (26.45-35.11)	<0.001
**Age**	0.24 (0.07-0.41)	0.005
**Female**	9.69 (5.79-13.59)	<0.001
Charlson comorbidity score items		
**Myocardial infarction**	18.48 (15.34-21.62)	<0.001
**Chronic heart failure**	38.06 (35.20-40.93)	<0.001
**Peripheral vascular disease**	29.19 (25.31-33.07)	<0.001
**Cerebrovascular disease**	32.49 (27.18-37.80)	<0.001
**Dementia**	40.73 (19.17-62.29)	<0.001
**Chronic pulmonary disease**	40.18 (31.41-48.95)	<0.001
**Rheumatic disease**	3.69 (-15.87-23.25)	0.711
**Peptic ulcer disease**	205.27 (170.99-239.54)	<0.001
**Mild liver disease**	37.52 (25.37-49.68)	<0.001
**Moderate to severe liver disease**	159.07 (125.07-193.08)	<0.001
**Diabetes without complications**	2.68 (-0.62-5.97)	0.111
**Diabetes with complications**	5.63 (-1.74-13.00)	0.134
**Paraplegia or hemiplegia**	87.90 (77.58-98.22)	<0.001
**Renal disease**	33.62 (29.30-37.94)	<0.001
**Cancer**	13.34 (-2.17-28.86)	0.092
**Metastatic cancer**	90.93 (11.09-170.77)	0.026
**AIDS**	42.84 (-32.08-117.76)	0.262
