# Supplementary material for: Does Chronic Obstructive Pulmonary Disease Impact Outcome after Coronary Artery Bypass Grafting? A Population-Based Retrospective Study in Germany
Source: J Clin Med. 2024 Aug 29;13(17):5131. doi: 10.3390/jcm13175131 (PMC11396234; doi:10.3390/jcm13175131)
Supplement: Supplementary file 1 [file jcm-13-05131-s001.zip › Additional File 11_Regression_copd_minimally invasive technique_mortality.pdf]

Additional File 11. Risk-adjusted associations of **in-hospital mortality** from multivariable regression analysis models analyzing the impact of cardiopulmonary bypass (CPB) in minimally invasive technique in 3,062 patients suffering from chronic obstructive pulmonary disease (COPD).

	**Odds ratio (95% CI)**	**P- value**
**CPB**	4.80 (2.42-9.51)	<0.001
**Age**	1.06 (1.04-1.09)	<0.001
**Female**	1.74 (1.13-2.67)	0.012
	Charlson comorbidity score items	
**Myocardial infarction**	1.87 (1.27-2.75)	0.002
**Chronic heart failure**	2.31 (1.44-3.70)	0.001
**Peripheral vascular disease**	2.29 (1.53-3.41)	<0.001
**Cerebrovascular disease**	0.99 (0.60-1.61)	0.960
**Dementia**	3.79 (0.77-18.62)	0.101
**Chronic pulmonary disease**	XXX	XXX
**Rheumatic disease**	0.42 (0.05-3.21)	0.400
**Peptic ulcer disease**	2.37 (0.60-9.34)	0.219
**Mild liver disease**	2.97 (1.30-6.77)	0.010
**Moderate to severe liver disease**	XXX	
**Diabetes without complications**	0.97 (0.63-1.48)	0.884
**Diabetes with complications**	0.65 (0.31-1.38)	0.263
**Paraplegia or hemiplegia**	0.25 (0.03-1.91)	0.184
**Renal disease**	1.62 (1.07-2.45)	0.022
**Cancer**	1.55 (0.56-4.30)	0.404
**Metastatic cancer**	2.10 (0.12-38.48)	0.612
**AIDS**	XXX	XXX

XXX: Omitted
