# Supplementary material for: Does Chronic Obstructive Pulmonary Disease Impact Outcome after Coronary Artery Bypass Grafting? A Population-Based Retrospective Study in Germany
Source: J Clin Med. 2024 Aug 29;13(17):5131. doi: 10.3390/jcm13175131 (PMC11396234; doi:10.3390/jcm13175131)
Supplement: Supplementary file 1 [file jcm-13-05131-s001.zip › Additional File 12_Regression_copd_minimally invasive technique_HLOS.pdf]

Additional File 12. Risk-adjusted associations of **hospital length of stay** from multivariable regression analysis models analyzing the impact of cardiopulmonary bypass (CPB) in minimally invasive technique in 3,065 patients suffering from chronic obstructive pulmonary disease (COPD).

	**Coefficient (95% CI)**	**P- value**
**CPB**	1.03 (-2.13-4.19)	0.522
**Age**	0.03 (-0.03-0.10)	0.348
**Female**	1.61 (0.37-2.86)	0.011
	Charlson comorbidity score items	
**Myocardial infarction**	0.80 (-0.32-1.92)	0.160
**Chronic heart failure**	2.39 (1.55-3.22)	<0.001
**Peripheral vascular disease**	1.02 (-0.50-2.08)	0.062
**Cerebrovascular disease**	1.46 (0.05-2.86)	0.043
**Dementia**	13.44 (2.71-24.18)	0.014
**Chronic pulmonary disease**	XXX	XXX
**Rheumatic disease**	-0.50 (-4.23-3.23)	0.793
**Peptic ulcer disease**	15.32 (5.83-24.82)	0.002
**Mild liver disease**	2.63 (-0.70-5.96)	0.122
**Moderate to severe liver disease**	0.42 (-17.76-18.59)	0.964
**Diabetes without complications**	1.57 (0.34-2.80)	0.013
**Diabetes with complications**	0.09 (-1.81-1.99)	0.926
**Paraplegia or hemiplegia**	10.41 (4.41-16.40)	0.001
**Renal disease**	2.64 (0.84-4.43)	0.004
**Cancer**	1.83 (-1.45-5.01)	0.260
**Metastatic cancer**	0.35 (-4.28-4.98)	0.881
**AIDS**	XXX	XXX

XXX: Omitted
