# Supplementary material for: Does Chronic Obstructive Pulmonary Disease Impact Outcome after Coronary Artery Bypass Grafting? A Population-Based Retrospective Study in Germany
Source: J Clin Med. 2024 Aug 29;13(17):5131. doi: 10.3390/jcm13175131 (PMC11396234; doi:10.3390/jcm13175131)
Supplement: Supplementary file 1 [file jcm-13-05131-s001.zip › Additional File 13_Regression_copd_minimally invasive technique_VT.pdf]

Additional File 13. Risk-adjusted associations of **perioperative ventilation time** from multivariable regression analysis models analyzing the impact of cardiopulmonary bypass (CPB) in minimally invasive technique in 1,157 patients suffering from chronic obstructive pulmonary disease (COPD).

	**Coefficient (95% CI)**	**P- value**
**CPB**	51.88 (-23.66-127.42)	0.178
**Age**	0.43 (-1.25-2.12)	0.615
**Female**	0.34 (-31.89-32.57)	0.983
Charlson comorbidity score items		
**Myocardial infarction**	15.00 (-13.33-43.32)	0.299
**Chronic heart failure**	48.18 (24.70-71.65)	<0.001
**Peripheral vascular disease**	-7.72 (-34.55-19.10)	0.572
**Cerebrovascular disease**	36.56 (-7.91-81.03)	0.107
**Dementia**	101.58 (-34.08-237.24)	0.142
**Chronic pulmonary disease**	XXX	XXX
**Rheumatic disease**	63.78 (-64.71-192.28)	0.330
**Peptic ulcer disease**	241.72 (57.63-425.81)	0.010
**Mild liver disease**	-17.84 (-62.77-27.10)	0.436
**Moderate to severe liver disease**	261.41 (180.07-342.75)	<0.001
**Diabetes without complications**	7.93 (-23.14-39.01)	0.617
**Diabetes with complications**	-2.66 (-43.43-38.11)	0.898
**Paraplegia or hemiplegia**	107.18 (12.37-201.99)	0.027
**Renal disease**	40.93 (-0.01-81.87)	0.050
**Cancer**	-66.58 (-95.48- -37.68)	<0.001
**Metastatic cancer**	10.34 (-190.07-210.74)	0.919
**AIDS**	XXX	XXX

XXX: Omitted
