# Supplementary material for: Does Chronic Obstructive Pulmonary Disease Impact Outcome after Coronary Artery Bypass Grafting? A Population-Based Retrospective Study in Germany
Source: J Clin Med. 2024 Aug 29;13(17):5131. doi: 10.3390/jcm13175131 (PMC11396234; doi:10.3390/jcm13175131)
Supplement: Supplementary file 1 [file jcm-13-05131-s001.zip › Additional File 14_Regression_no copd_minimally invasive technique_mortality.pdf]

Additional File 14. Risk-adjusted associations of **in-hospital mortality** from multivariable regression analysis models analyzing the impact of cardiopulmonary bypass (CPB) in minimally invasive technique in 40,380 patients not suffering from chronic obstructive pulmonary disease (COPD).

	**Odds ratio (95% CI)**	**P- value**
**CPB**	6.73 (5.50-8.25)	<0.001
**Age**	1.04 (1.03-1.05)	<0.001
**Female**	1.58 (1.36-1.84)	<0.001
Charlson comorbidity score items		
**Myocardial infarction**	2.48 (2.16-2.85)	<0.001
**Chronic heart failure**	2.26 (1.94-2.63)	<0.001
**Peripheral vascular disease**	1.90 (1.64-2.21)	<0.001
**Cerebrovascular disease**	1.48 (1.23-1.77)	<0.001
**Dementia**	1.89 (1.04-3.44)	0.037
**Chronic pulmonary disease**	1.95 (1.48-2.58)	<0.001
**Rheumatic disease**	1.17 (0.66-2.10)	0.592
**Peptic ulcer disease**	3.90 (2.33-6.52)	<0.001
**Mild liver disease**	1.91 (1.26-2.88)	0.002
**Moderate to severe liver disease**	13.68 (6.93-27.04)	<0.001
**Diabetes without complications**	0.84 (0.72-0.99)	0.032
**Diabetes with complications**	0.94 (0.73-1.21)	0.628
**Paraplegia or hemiplegia**	1.61 (1.17-2.23)	0.004
**Renal disease**	1.44 (1.22-1.70)	<0.001
**Cancer**	1.99 (1.25-3.16)	0.003
**Metastatic cancer**	2.53 (0.92-6.99)	0.073
**AIDS**	XXX	XXX

XXX: Omitted
