# Supplementary material for: Does Chronic Obstructive Pulmonary Disease Impact Outcome after Coronary Artery Bypass Grafting? A Population-Based Retrospective Study in Germany
Source: J Clin Med. 2024 Aug 29;13(17):5131. doi: 10.3390/jcm13175131 (PMC11396234; doi:10.3390/jcm13175131)
Supplement: Supplementary file 1 [file jcm-13-05131-s001.zip › Additional File 15_Regression_ no copd_minimally invasive technique_HLOS.pdf]

Additional File 15. Risk-adjusted associations of **hospital length of stay** from multivariable regression analysis models analyzing the impact of cardiopulmonary bypass (CPB) in minimally invasive technique in 40,393 patients not suffering from chronic obstructive pulmonary disease (COPD).

	**Coefficient (95% CI)**	**P- value**
**CPB**	3.21 (2.33-4.10)	<0.001
**Age**	0.06 (0.05-0.07)	<0.001
**Female**	1.13 (0.87-1.39)	<0.001
Charlson comorbidity score items		
**Myocardial infarction**	0.33 (0.12-0.53)	0.002
**Chronic heart failure**	1.31 (1.13-1.47)	<0.001
**Peripheral vascular disease**	1.00 (0.70-1.30)	<0.001
**Cerebrovascular disease**	1.72 (1.31-2.13)	<0.001
**Dementia**	2.74 (1.19-4.30)	0.001
**Chronic pulmonary disease**	2.27 (1.55-2.98)	<0.001
**Rheumatic disease**	0.99 (-0.76-2.74)	0.268
**Peptic ulcer disease**	22.13 (15.74-28.53)	<0.001
**Mild liver disease**	1.52 (0.57-2.48)	0.002
**Moderate to severe liver disease**	8.89 (-0.03-17.82)	0.051
**Diabetes without complications**	0.73 (0.53-0.93)	<0.001
**Diabetes with complications**	2.32 (1.69-2.94)	<0.001
**Paraplegia or hemiplegia**	5.93 (4.43-7.43)	<0.001
**Renal disease**	1.58 (1.24-1.92)	<0.001
**Cancer**	2.85 (1.64-4.06)	<0.001
**Metastatic cancer**	3.09 (-0.03-6.22)	0.052
**AIDS**	-2.52 (-4.18- -0.85)	0.003
