# Supplementary material for: Does Chronic Obstructive Pulmonary Disease Impact Outcome after Coronary Artery Bypass Grafting? A Population-Based Retrospective Study in Germany
Source: J Clin Med. 2024 Aug 29;13(17):5131. doi: 10.3390/jcm13175131 (PMC11396234; doi:10.3390/jcm13175131)
Supplement: Supplementary file 1 [file jcm-13-05131-s001.zip › Additional File 16_Regression_ no copd_minimally invasive technique_VT.pdf]

Additional File 16. Risk-adjusted associations of **perioperative ventilation time** from multivariable regression analysis models analyzing the impact of cardiopulmonary bypass (CPB) in minimally invasive technique in 10,454 patients not suffering from chronic obstructive pulmonary disease (COPD).

	**Coefficient (95% CI)**	**P- value**
**CPB**	111.80 (87.85-135.76)	<0.001
**Age**	0.36 (-0.04-0.77)	0.080
**Female**	15.19 (3.98-26.38)	0.008
	Charlson comorbidity score items	
**Myocardial infarction**	21.37 (12.88-29.86)	<0.001
**Chronic heart failure**	21.24 (13.73-28.76)	<0.001
**Peripheral vascular disease**	14.43 (3.39-25.46)	0.010
**Cerebrovascular disease**	65.07 (44.03-86.12)	<0.001
**Dementia**	21.80 (-16.63-60.23)	0.266
**Chronic pulmonary disease**	53.46 (25.49-81.42)	<0.001
**Rheumatic disease**	67.35 (-74.40-209.11)	0.352
**Peptic ulcer disease**	372.68 (195.85-549.51)	<0.001
**Mild liver disease**	-6.29 (-31.07-18.49)	0.619
**Moderate to severe liver disease**	141.97 (1.20-282.75)	0.048
**Diabetes without complications**	2.05 (-6.66-10.77)	0.644
**Diabetes with complications**	7.50 (-10.43-25.43)	0.412
**Paraplegia or hemiplegia**	49.06 (16.41-81.70)	0.003
**Renal disease**	19.00 (6.24-31.75)	0.004
**Cancer**	30.12 (-4.71-64.95)	0.090
**Metastatic cancer**	-18.51 (-61.26-24.24)	0.396
**AIDS**	XXX	XXX

XXX: Omitted
