# Supplementary material for: Does Chronic Obstructive Pulmonary Disease Impact Outcome after Coronary Artery Bypass Grafting? A Population-Based Retrospective Study in Germany
Source: J Clin Med. 2024 Aug 29;13(17):5131. doi: 10.3390/jcm13175131 (PMC11396234; doi:10.3390/jcm13175131)
Supplement: Supplementary file 1 [file jcm-13-05131-s001.zip › Additional file 1_ICD_OPS_rev.pdf]

Additional file 1. Details of variable transcoding for procedures and diagnoses (OPS procedural code and ICD 10- classification). Details of data source.

Coronary artery bypass grafting	
Coronary artery bypass grafting on-pump	5361, 5362 + 8851
Coronary artery bypass grafting off-pump	5362
**Chronic obstructive pulmonary disease**	
Chronic obstructive pulmonary disease	J448, J449
**Comorbidities (Charlson Comorbidity Index)**	
Acute myocardial infarction	I21, I22, I252
Congestive heart failure	I43, I50, I099, I110, I130, I132, I255, I420, I425, I426, I427, I428, I429, P290
Peripheral vascular disease	I70, I71, I731, I738, I739, I771, I790, I792, K551, K558, K559, Z958, Z959
Cerebrovascular disease	G45, G46, I60, I61, I62, I63, I64, I65, I66, I67, I68, I69, H340
Dementia	F00, F01, F02, F03, G30, F051, G311
Chronic pulmonary disease	J40, J41, J42, J43, J44, J45, J46, J47, J60, J61, J62, J63, J64, J65, J66, J67, I278, I279, J684, J701, J703
Rheumatologic disease	M05, M32, M33, M34, M06, M315, M351, M353, M360
Peptic ulcer disease	K25, K26, K27, K28
Mild liver disease	B18, K73, K74, K700, K701, K702, K703, K709, K713, K714, K715, K717, K760, K762, K763, K764, K768, K769, Z944
Moderate/severe liver disease	K704, K711, K721, K729, K765, K766, K767, I850, I859, I864, I982
Diabetes without complications	E100, E101, E106, E108, E109, E110, E111, E116, E118, E119, E120, E121, E126, E128, E129, E130, E131, E136, E138, E139, E140, E141, E146, E148, E149
Diabetes with chronic complications	E102, E103, E104, E105, E107, E112, E113, E114, E115, E117, E122, E123, E124, E125, E127, E132, E133, E134, E135, E137, E142, E143, E144, E145, E147
Hemiplegia or paraplegia	G81, G82, G041, G114, G801, G802, G830, G831, G832, G833, G834, G839
Renal disease	N18, N19, Z49, N052, N053, N054, N055, N056, N057, N250, I120, I131, N032, N033, N034, N035, N036, N037, Z490, Z491, Z492, Z940, Z992
Cancer	C00, C01, C02, C03, C04, C05, C06, C07, C08, C09, C10, C11, C12, C13, C14, C15, C16, C17, C18, C19, C20, C21, C22, C23, C24, C25, C26, C30, C31, C32, C33, C34, C37, C38, C39, C40, C41, C43, C45, C46, C47, C48, C49, C50, C51, C52, C53, C54, C55, C56, C57, C58, C60, C61, C62, C63, C64, C65, C66, C67,

	C68, C69, C70, C71, C72, C73, C74, C75, C76, C81, C82, C83, C84, C85, C88, C90, C91, C92, C93, C94, C95, C96, C97
Metastatic cancer	C77, C78, C79, C80
AIDS/HIV	B20, B21, B22, B23, B24, U60, U61, Z21
Data source	
RDC of the Federal Statistical Office and Statistical Offices of the Federal States, Source DOI (own calculations)	10.21242/23141.2021.00.00.1.1.0, 10.21242/23141.2020.00.00.1.1.0, 10.21242/23141.2019.00.00.1.1.1, 10.21242/23141.2018.00.00.1.1.0, 10.21242/23141.2017.00.00.1.1.0, 10.21242/23141.2016.00.00.1.1.0, 10.21242/23141.2015.00.00.1.1.0
