# Supplementary material for: Does Chronic Obstructive Pulmonary Disease Impact Outcome after Coronary Artery Bypass Grafting? A Population-Based Retrospective Study in Germany
Source: J Clin Med. 2024 Aug 29;13(17):5131. doi: 10.3390/jcm13175131 (PMC11396234; doi:10.3390/jcm13175131)
Supplement: Supplementary file 1 [file jcm-13-05131-s001.zip › Additional File 2_Regression_copd_mortality.pdf]

Additional File 2. Risk-adjusted associations of **in-hospital mortality** from multivariable regression analysis models analyzing the impact of chronic obstructive pulmonary disease (COPD) in 274,792 patients undergoing coronary artery bypass grafting (CABG).

	**Odds ratio (95% CI)**	**P- value**
**COPD**	0.94 (0.84-1.04)	0.214
**Age**	1.04 (1.03-1.04)	<0.001
**Female**	1.66 (1.59-1.73)	<0.001
Charlson comorbidity score items		
**Myocardial infarction**	1.86 (1.79-1.93)	<0.001
**Chronic heart failure**	2.63 (2.52-2.75)	<0.001
**Peripheral vascular disease**	2.02 (1.94-2.10)	<0.001
**Cerebrovascular disease**	1.38 (1.32-1.45)	<0.001
**Dementia**	1.22 (1.01-1.49)	0.044
**Chronic pulmonary disease**	1.23 (1.13-1.45)	<0.001
**Rheumatic disease**	0.95 (0.81-1.12)	0.537
**Peptic ulcer disease**	2.53 (2.17-2.94)	<0.001
**Mild liver disease**	2.34 (2.12-2.58)	<0.001
**Moderate to severe liver disease**	10.76 (9.08-12.76)	<0.001
**Diabetes without complications**	0.95 (0.91-1.00)	0.030
**Diabetes with complications**	0.92 (0.85-0.99)	0.022
**Paraplegia or hemiplegia**	1.33 (1.21-1.45)	<0.001
**Renal disease**	1.51 (1.45-1.58)	<0.001
**Cancer**	1.30 (1.13-1.50)	<0.001
**Metastatic cancer**	1.95 (1.37-2.78)	<0.001
**AIDS**	1.17 (0.36-3.80)	0.789
