# Supplementary material for: Does Chronic Obstructive Pulmonary Disease Impact Outcome after Coronary Artery Bypass Grafting? A Population-Based Retrospective Study in Germany
Source: J Clin Med. 2024 Aug 29;13(17):5131. doi: 10.3390/jcm13175131 (PMC11396234; doi:10.3390/jcm13175131)
Supplement: Supplementary file 1 [file jcm-13-05131-s001.zip › Additional File 3_Regression_copd_HLOS.pdf]

Additional File 3. Risk-Adjusted associations of **hospital length of stay** from multivariable regression analysis models analyzing the impact of chronic obstructive pulmonary disease (COPD) in 274,792 patients undergoing coronary artery bypass grafting (CABG).

	**Coefficient (95% CI)**	**P- value**
**COPD**	-0.73 (-1.07- -0.38)	<0.001
**Age**	0.06 (0.06-0.07)	<0.001
**Female**	1.19 (1.08-1.31)	<0.001
Charlson comorbidity score items		
**Myocardial infarction**	0.03 (-0.06-0.12)	0.447
**Chronic heart failure**	1.86 (1.77-1.94)	<0.001
**Peripheral vascular disease**	1.53 (1.41-1.65)	<0.001
**Cerebrovascular disease**	1.35 (1.20-1.51)	<0.001
**Dementia**	3.91 (3.03-4.79)	<0.001
**Chronic pulmonary disease**	2.45 (2.16-2.75)	<0.001
**Rheumatic disease**	1.12 (0.67-1.57)	<0.001
**Peptic ulcer disease**	14.61 (13.27-15.95)	<0.001
**Mild liver disease**	2.28 (1.84-2.73)	<0.001
**Moderate to severe liver disease**	7.74 (5.92-9.56)	<0.001
**Diabetes without complications**	0.71 (0.61-0.80)	<0.001
**Diabetes with complications**	2.35 (2.08-2.62)	<0.001
**Paraplegia or hemiplegia**	6.81 (6.33-7.29)	<0.001
**Renal disease**	2.48 (2.34-2.62)	<0.001
**Cancer**	2.79 (2.22-3.36)	<0.001
**Metastatic cancer**	7.89 (5.43-10.34)	<0.001
**AIDS**	0.03 (-1.75-1.81)	0.974
