# Supplementary material for: Does Chronic Obstructive Pulmonary Disease Impact Outcome after Coronary Artery Bypass Grafting? A Population-Based Retrospective Study in Germany
Source: J Clin Med. 2024 Aug 29;13(17):5131. doi: 10.3390/jcm13175131 (PMC11396234; doi:10.3390/jcm13175131)
Supplement: Supplementary file 1 [file jcm-13-05131-s001.zip › Additional File 4_Regression_ copd_VT.pdf]

Additional File 4. Risk-Adjusted associations of **perioperative ventilation time** from multivariable regression analysis models analyzing the impact of chronic obstructive pulmonary disease (COPD) in 91,611 patients undergoing coronary artery bypass grafting (CABG).

	Coefficient (95% CI)	P- value
**COPD**	-24.32 (-34.46- -14.17)	<0.001
**Age**	0.27 (0.12-0.43)	0.001
**Female**	8.42 (4.72-12.13)	<0.001
	Charlson comorbidity score items	
**Myocardial infarction**	18.03 (15.00-21.06)	<0.001
**Chronic heart failure**	38.62 (35.81-41.42)	<0.001
**Peripheral vascular disease**	29.09 (25.39-32.78)	<0.001
**Cerebrovascular disease**	32.00 (26.94-37.06)	<0.001
**Dementia**	40.44 (20.16-60.71)	<0.001
**Chronic pulmonary disease**	40.41 (-(31.65-49.18)	<0.001
**Rheumatic disease**	4.42 (-13.55-22.39)	0.630
**Peptic ulcer disease**	205.34 (173.18-237.49)	<0.001
**Mild liver disease**	39.03 (27.26-50.79)	<0.001
**Moderate to severe liver disease**	165.08 (132.56-197.60)	<0.001
**Diabetes without complications**	3.09 (-0.10-6.27)	0.057
**Diabetes with complications**	7.88 (0.52-15.24)	0.036
**Paraplegia or hemiplegia**	91.05 (81.17-100.92)	<0.001
**Renal disease**	35.63 (31.47-39.79)	<0.001
**Cancer**	17.92 (0.86-34.88)	0.038
**Metastatic cancer**	85.42 (11.23-159.61)	0.024
**AIDS**	46.25 (-28.50-120.99)	0.225
