# Supplementary material for: Does Chronic Obstructive Pulmonary Disease Impact Outcome after Coronary Artery Bypass Grafting? A Population-Based Retrospective Study in Germany
Source: J Clin Med. 2024 Aug 29;13(17):5131. doi: 10.3390/jcm13175131 (PMC11396234; doi:10.3390/jcm13175131)
Supplement: Supplementary file 1 [file jcm-13-05131-s001.zip › Additional File 5_Regression_copd_on-pump_mortality.pdf]

Additional File 5. Risk-adjusted associations of **in-hospital mortality** from multivariable regression analysis models analyzing the impact of on-pump aorto-coronary bypass surgery in 21,238 patients suffering from chronic obstructive pulmonary disease (COPD).

	**Odds ratio (95% CI)**	**P- value**
**On-pump surgery**	1.86 (1.51-2.29)	<0.001
**Age**	1.05 (1.04-1.06)	<0.001
**Female**	1.65 (1.45-1.88)	<0.001
	Charlson comorbidity score items	
**Myocardial infarction**	1.59 (1.41-1.79)	<0.001
**Chronic heart failure**	2.58 (2.23-2.99)	<0.001
**Peripheral vascular disease**	1.67 (1.48-1.88)	<0.001
**Cerebrovascular disease**	1.41 (1.23-1.62)	<0.001
**Dementia**	1.18 (0.68-2.06)	0.553
**Chronic pulmonary disease**	XXX	XXX
**Rheumatic disease**	0.90 (0.56-1.42)	0.641
**Peptic ulcer disease**	1.60 (0.96-2.64)	0.069
**Mild liver disease**	2.54 (1.97-3.27)	<0.001
**Moderate to severe liver disease**	5.51 (3.10-9.80)	<0.001
**Diabetes without complications**	0.94 (0.83-1.07)	0.379
**Diabetes with complications**	0.90 (0.73-1.12)	0.349
**Paraplegia or hemiplegia**	1.24 (0.95-1.61)	0.113
**Renal disease**	1.54 (1.36-1.75)	<0.001
**Cancer**	1.73 (1.21-2.47)	0.003
**Metastatic cancer**	1.32 (0.37-4.68)	0.665
**AIDS**	XXX	

XXX: Omitted
