# Supplementary material for: Does Chronic Obstructive Pulmonary Disease Impact Outcome after Coronary Artery Bypass Grafting? A Population-Based Retrospective Study in Germany
Source: J Clin Med. 2024 Aug 29;13(17):5131. doi: 10.3390/jcm13175131 (PMC11396234; doi:10.3390/jcm13175131)
Supplement: Supplementary file 1 [file jcm-13-05131-s001.zip › Additional File 6_Regression_copd_on pump_HLOS.pdf]

Additional File 6. Risk-Adjusted associations of **hospital length of stay** from multivariable regression analysis models analyzing the impact of on-pump aorto-coronary bypass surgery in 21,240 patients suffering from chronic obstructive pulmonary disease (COPD).

	**Coefficient (95% CI)**	**P- value**
**On-pump surgery**	1.44 (0.91-1.97)	<0.001
**Age**	0.06 (0.03-0.08)	<0.001
**Female**	0.81 (0.34-1.28)	0.001
Charlson comorbidity score items		
**Myocardial infarction**	-0.05 (-0.45-0.36)	0.812
**Chronic heart failure**	2.31 (1.95-2.67)	<0.001
**Peripheral vascular disease**	1.30 (0.85-1.74)	<0.001
**Cerebrovascular disease**	0.86 (0.29-1.43)	0.003
**Dementia**	4.10 (1.23-6.93)	0.005
**Chronic pulmonary disease**	XXX	
**Rheumatic disease**	0.21 (-1.52-1.94)	0.812
**Peptic ulcer disease**	14.58 (10.42-18.74)	<0.001
**Mild liver disease**	2.22 (0.67-3.78)	0.005
**Moderate to severe liver disease**	11.09 (5.10-17.07)	<0.001
**Diabetes without complications**	1.30 (0.87-1.72)	<0.001
**Diabetes with complications**	3.05 (1.98-4.12)	<0.001
**Paraplegia or hemiplegia**	8.21 (6.52-9.91)	<0.001
**Renal disease**	3.10 (2.54-7.69)	<0.001
**Cancer**	5.11 (2.53-7.69)	<0.001
**Metastatic cancer**	9.56 (3.76-15.35)	0.001
**AIDS**	-6.09 (-9.00- -3.18)	<0.001

XXX: Omitted
