# Supplementary material for: Does Chronic Obstructive Pulmonary Disease Impact Outcome after Coronary Artery Bypass Grafting? A Population-Based Retrospective Study in Germany
Source: J Clin Med. 2024 Aug 29;13(17):5131. doi: 10.3390/jcm13175131 (PMC11396234; doi:10.3390/jcm13175131)
Supplement: Supplementary file 1 [file jcm-13-05131-s001.zip › Additional File 7_Regression_copd_on pump_VT.pdf]

Additional File 7. Risk-Adjusted associations of **perioperative ventilation time** from multivariable regression analysis models analyzing the impact of on-pump aorto-coronary bypass surgery in 9,103 patients suffering from chronic obstructive pulmonary disease (COPD).

	**Coefficient (95% CI)**	**P- value**
**On-pump surgery**	33.67 (18.67-48.66)	<0.001
**Age**	0.48 (-0,15-1.10)	0.135
**Female**	-5.05 (-16.98-6.87)	0.406
Charlson comorbidity score items		
**Myocardial infarction**	11.35 (0.34-22.38)	0.043
**Chronic heart failure**	37.51 (26.39-48.63)	<0.001
**Peripheral vascular disease**	22.20 (10.64-33.75)	<0.001
**Cerebrovascular disease**	19.30 (2.89-35.71)	0.021
**Dementia**	39.79 (-19.66-99.24)	0.190
**Chronic pulmonary disease**	XXX	XXX
**Rheumatic disease**	3.16 (-41.11-47.43)	0.889
**Peptic ulcer disease**	202.24 (108.16-296.33)	<0.001
**Mild liver disease**	49.29 (10.03-88.54)	0.014
**Moderate to severe liver disease**	213.42 (102.80-324.05)	<0.001
**Diabetes without complications**	8.91 (-2.40-20.21)	0.122
**Diabetes with complications**	27.22 (-1.54-55.97)	0.064
**Paraplegia or hemiplegia**	112.58 (80.29-144.87)	<0.001
**Renal disease**	47.65 (33.35-61.96)	<0.001
**Cancer**	53.64 (-28.48-135.76)	0.200
**Metastatic cancer**	28.01 (-116.92-172.94)	0.705
**AIDS**	XXX	XXX

XXX: Omitted
