# Supplementary material for: Does Chronic Obstructive Pulmonary Disease Impact Outcome after Coronary Artery Bypass Grafting? A Population-Based Retrospective Study in Germany
Source: J Clin Med. 2024 Aug 29;13(17):5131. doi: 10.3390/jcm13175131 (PMC11396234; doi:10.3390/jcm13175131)
Supplement: Supplementary file 1 [file jcm-13-05131-s001.zip › Additional File 8_Regression_no copd_on-pump_mortality.pdf]

Additional File 8. Risk-adjusted associations of **in-hospital mortality** from multivariable regression analysis models analyzing the impact of on-pump aorto-coronary bypass surgery in 253,552 patients not suffering from chronic obstructive pulmonary disease (no-COPD).

	**Odds ratio (95% CI)**	**P- value**
**On-pump surgery**	2.19 (2.03-2.37)	<0.001
**Age**	1.04 (1.03-1.04)	<0.001
**Female**	1.66 (1.59-1.74)	<0.001
Charlson comorbidity score items		
**Myocardial infarction**	1.90 (1.82-1.98)	<0.001
**Chronic heart failure**	2.61 (2.49-2.73)	<0.001
**Peripheral vascular disease**	2.05 (1.97-2.14)	<0.001
**Cerebrovascular disease**	1.37 (1.30-1.44)	<0.001
**Dementia**	1.22 (0.99-1.50)	0.068
**Chronic pulmonary disease**	1.22 (1.12-1.33)	<0.001
**Rheumatic disease**	0.94 (0.79-1.12)	0.509
**Peptic ulcer disease**	2.62 (2.24-3.08)	<0.001
**Mild liver disease**	2.30 (2.08-2.56)	<0.001
**Moderate to severe liver disease**	11.23 (9.39-13.43)	<0.001
**Diabetes without complications**	0.96 (0.91-1.00)	0.053
**Diabetes with complications**	0.93 (0.86-1.01)	0.077
**Paraplegia or hemiplegia**	1.31 (1.19-1.44)	<0.001
**Renal disease**	1.50 (1.43-1.57)	<0.001
**Cancer**	1.25 (1.07-1.46)	0.006
**Metastatic cancer**	2.09 (1.45-3.01)	<0.001
**AIDS**	1.21 (0.37-3.93)	0.755
