# Supplementary material for: Does Chronic Obstructive Pulmonary Disease Impact Outcome after Coronary Artery Bypass Grafting? A Population-Based Retrospective Study in Germany
Source: J Clin Med. 2024 Aug 29;13(17):5131. doi: 10.3390/jcm13175131 (PMC11396234; doi:10.3390/jcm13175131)
Supplement: Supplementary file 1 [file jcm-13-05131-s001.zip › Additional File 9_Regression_ no copd_on pump_HLOS.pdf]

Additional File 9. Risk-Adjusted associations of **hospital length of stay** from multivariable regression analysis models analyzing the impact of on-pump aorto-coronary bypass surgery in 253,552 patients not suffering from chronic obstructive pulmonary disease (no-COPD).

	**Coefficient (95% CI)**	**P- value**
**On-pump surgery**	1.61 (1.51-1.71)	<0.001
**Age**	0.06 (0.06-0.07)	<0.001
**Female**	1.22 (1.10-1.34)	<0.001
Charlson comorbidity score items		
**Myocardial infarction**	0.03 (-0.06-0.12)	0.519
**Chronic heart failure**	1.78 (1.70-1.86)	<0.001
**Peripheral vascular disease**	1.52 (1.39-1.64)	<0.001
**Cerebrovascular disease**	1.37 (1.21-1.53)	<0.001
**Dementia**	3.87 (2.95-4.79)	<0.001
**Chronic pulmonary disease**	2.42 (2.13-2.72)	<0.001
**Rheumatic disease**	1.16 (0.70-1.63)	<0.001
**Peptic ulcer disease**	14.56 (13.14-15.98)	<0.001
**Mild liver disease**	2.23 (1.77-2.70)	<0.001
**Moderate to severe liver disease**	7.28 (5.37-9.19)	<0.001
**Diabetes without complications**	0.65 (0.56-0.74)	<0.001
**Diabetes with complications**	2.31 (2.04-2.59)	<0.001
**Paraplegia or hemiplegia**	6.61 (6.11-7.11)	<0.001
**Renal disease**	2.38 (2.24-2.53)	<0.001
**Cancer**	2.50 (1.95-3.06)	<0.001
**Metastatic cancer**	7.79 (5.17-10.39)	<0.001
**AIDS**	0.17 (-1.61-1.96)	0.849
